# Young-onset diabetes in Asian Indians is associated with lower measured and genetically determined beta cell function

**DOI:** 10.1007/s00125-022-05671-z

**Published:** 2022-03-05

**Authors:** Moneeza K. Siddiqui, Ranjit Mohan Anjana, Adem Y. Dawed, Cyrielle Martoeau, Sundararajan Srinivasan, Jebarani Saravanan, Sathish K. Madanagopal, Alasdair Taylor, Samira Bell, Abirami Veluchamy, Rajendra Pradeepa, Naveed Sattar, Radha Venkatesan, Colin N. A. Palmer, Ewan R. Pearson, Viswanathan Mohan

**Affiliations:** 1 National Institute for Health Research Global Health Unit for Diabetes Outcomes Research, Division of Population Health & Genomics, School of Medicine, University of Dundee, Dundee, UK; 2 Dr Mohan’s Diabetes Specialities Centre and Madras Diabetes Research Foundation, Chennai, India; 3 Institute of Cardiovascular and Medical Sciences, University of Glasgow, Glasgow, UK

**Keywords:** Beta cell function, Epidemiology, Genetics of type 2 diabetes, South Asian diabetes, Young-onset diabetes

## Abstract

**Aims/hypothesis:**

South Asians in general, and Asian Indians in particular, have higher risk of type 2 diabetes compared with white Europeans, and a younger age of onset. The reasons for the younger age of onset in relation to obesity, beta cell function and insulin sensitivity are under-explored.

**Methods:**

Two cohorts of Asian Indians, the ICMR-INDIAB cohort (Indian Council of Medical Research-India Diabetes Study) and the DMDSC cohort (Dr Mohan’s Diabetes Specialties Centre), and one of white Europeans, the ESDC (East Scotland Diabetes Cohort), were used. Using a cross-sectional design, we examined the comparative prevalence of healthy, overweight and obese participants with young-onset diabetes, classified according to their BMI. We explored the role of clinically measured beta cell function in diabetes onset in Asian Indians. Finally, the comparative distribution of a partitioned polygenic score (pPS) for risk of diabetes due to poor beta cell function was examined. Replication of the genetic findings was sought using data from the UK Biobank.

**Results:**

The prevalence of young-onset diabetes with normal BMI was 9.3% amongst white Europeans and 24–39% amongst Asian Indians. In Asian Indians with young-onset diabetes, after adjustment for family history of type 2 diabetes, sex, insulin sensitivity and HDL-cholesterol, stimulated C-peptide was 492 pmol/ml (IQR 353–616, *p*<0.0001) lower in lean compared with obese individuals. Asian Indians in our study, and South Asians from the UK Biobank, had a higher number of risk alleles than white Europeans. After weighting the pPS for beta cell function, Asian Indians have lower genetically determined beta cell function than white Europeans (*p*<0.0001). The pPS was associated with age of diagnosis in Asian Indians but not in white Europeans. The pPS explained 2% of the variation in clinically measured beta cell function, and 1.2%, 0.97%, and 0.36% of variance in age of diabetes amongst Asian Indians with normal BMI, or classified as overweight and obese BMI, respectively.

**Conclusions/interpretation:**

The prevalence of lean BMI in young-onset diabetes is over two times higher in Asian Indians compared with white Europeans. This phenotype of lean, young-onset diabetes appears driven in part by lower beta cell function. We demonstrate that Asian Indians with diabetes also have lower genetically determined beta cell function.

**Graphical abstract:**

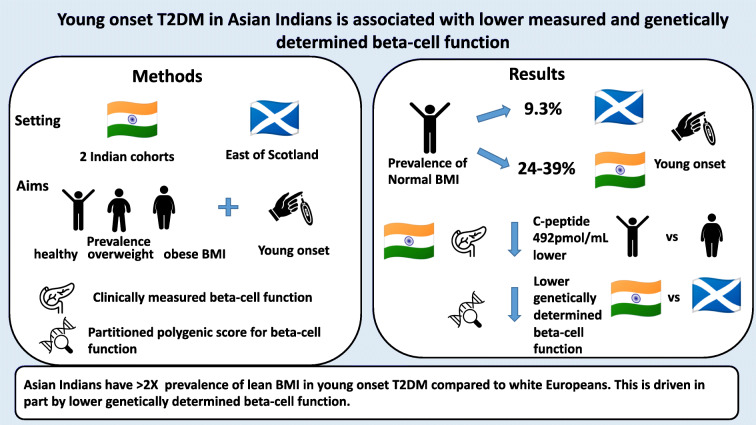

**Supplementary Information:**

The online version contains peer-reviewed but unedited supplementary material available at 10.1007/s00125-022-05671-z.

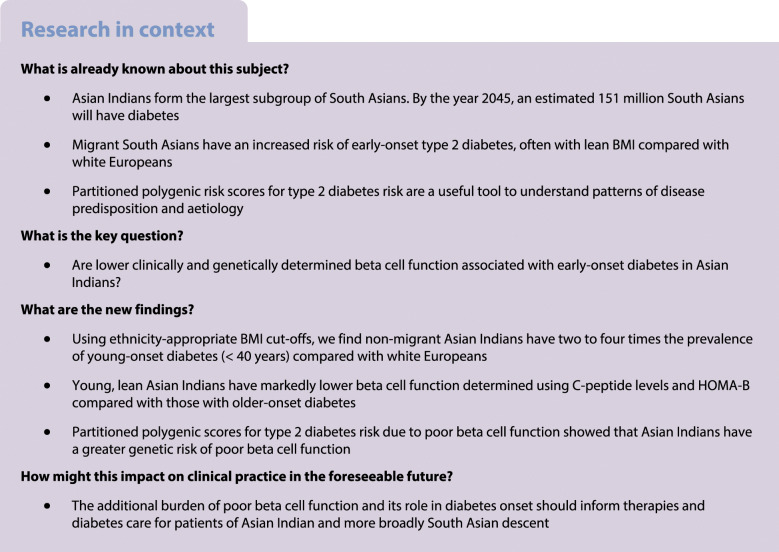



## Introduction

Diabetes prevalence is increasing, with a major burden of morbidity in South Asia. The International Diabetes Federation projects that by 2045 there will be 151.4 million indigenous South Asians with type 2 diabetes, almost doubling the present prevalence [1]. A large-scale population survey, the Indian Council for Medical Research-India Diabetes (ICMR-INDIAB) study, reported that the mean prevalence of diabetes in India was 7.3%, with substantial variation across states [2]. This is higher than the current prevalence in Scotland (6.3%), a representative white European population, or most of Europe according to International Diabetes Federation estimates. Type 2 diabetes in South Asians is a global problem, as they form a substantial part of the diaspora in Western countries [3, 4]. South Asians living in high-income countries are reported to be diagnosed with diabetes between 5 and 10 years earlier than white Europeans [5–8]. To reduce the global burden of diabetes, there is a need to better characterise the specific metabolic and physiological drivers of diabetes in this ethnic group.

The metabolic phenotype of Asian Indians differs markedly from the phenotype seen in white people. The phenotype of the ‘thin–fat’ Indian, best captured in the comparative dual energy x-ray absorptiometry scan conducted by Yajnik and Yudkin [9]. This phenotype is of increased truncal fat despite normal or low BMI, which is associated with hyperinsulinaemia and other measures of insulin resistance, and is seen from birth [10–13], in children [14, 15] and through adult life. A number of studies have investigated the association of this thin–fat phenotype with diabetes risk [16–19]. So far, large-scale studies have focused on migrant South Asian populations living in high-income countries. Of these, two comparative studies across ethnicities are of note. The SABRE study [19] was a prospective cohort study comparing risk for diabetes and variables associated with risk in white, Asian Indian and Afro-Caribbean ethnic groups living in the UK. They found, compared with white Europeans, Asian Indian men who went on to develop diabetes over a 20-year follow-up period have lower BMI, higher waist–hip ratio, higher truncal skinfold thickness, higher insulin resistance, and increased (compensatory) beta cell function, but no difference in lipids. In addition, an analysis of the UK Biobank established that, to have the same diabetes risk as white participants with a BMI >30 kg/m^2^, the equivalent BMI in South Asian participants was only 22 kg/m^2^ [20].

While it has been established that type 2 diabetes in Asian Indians is characterised by younger age of onset with a relatively low or lower BMI [21–23], no large-scale studies have been undertaken to comprehensively characterise the metabolic phenotype and genetic risk of young, lean Asian Indians who develop type 2 diabetes. A major challenge in comparative studies of diabetes across ethnicities is the lack of genetic resources that can be used to overcome biases introduced when comparing clinical measures from different geographic health settings. The India–Scotland Partnership for Precision Medicine in Diabetes (INSPIRED) brings together data resources for the study of diabetes in India and Scotland (UK). As far as we are aware, the present study is the first population study to comprehensively understand the metabolic risk factors for young-onset diabetes in an Asian Indian cohort using ethnicity-specific thresholds for obesity. We then tested the hypothesis that the phenotype of young-onset diabetes in Asian Indians is one of beta cell deficiency, using both clinical data and a partitioned or process-specific polygenic score (pPS) for type 2 diabetes susceptibility due to impaired beta cell function.

## Methods

### Cohorts used

The main inclusion criterion for the study for all cohorts was diagnosis of type 2 diabetes using the WHO criteria [24]. Data contribution by study cohort is explained in electronic supplementary material (ESM) Table 1 and ESM Methods: Clinical Variables.

#### ICMR-INDIAB cohort

The Asian Indian cohort is derived from published data of the ICMR-INDIAB study [2]. This is a national cross-sectional study to establish the national and state-specific prevalence of diabetes and prediabetes in India. The survey methodology has been described previously [25]. Individuals underwent an OGTT (which included a fasting and 2 h post 75 g load glucose measurement) [24], and were classified as having newly diagnosed diabetes according to the WHO criteria [24]. From this cohort, only age of diagnosis and BMI were considered.

#### INSPIRED: ESDC

The cohort includes white European individuals with type 2 diabetes from the East of Scotland (the East Scotland Diabetes Cohort [ESDC]), across the regions of Tayside and Fife. Clinical data are made available through the Scottish Care Information–Diabetes Collaboration (SCI–DC) system [26]. General practitioners confirm diagnosis using WHO criteria [24] for diagnosis on the basis of fasting or random glucose, OGTT or HbA_1c_ [24]. Participants included individuals with a recorded diagnosis and for whom all anthropometric and clinical measures were reported within 1 year of diagnosis. From this cohort, age of diagnosis and BMI were considered. Additionally, genotypic data were utilised.

#### INSPIRED: DMDSC

Clinical data from Asian Indians with diabetes was obtained from Dr. Mohan’s Diabetes Specialities Centre (DMDSC). This is a privately run chain of single-speciality hospitals and clinics for the treatment of diabetes and comorbidities, and is a key site for diabetes research in India [2, 27, 28]. Data linkage was performed using a unified electronic health system across clinic, biochemistry and other subspecialties. Physiological, biochemical and genetic factors associated with young lean-onset diabetes were examined in this cohort.

In addition to the inclusion criterion of diagnosis of type 2 diabetes using the WHO criteria [24], in the DMDSC cohort, all patients were excluded if clinically diagnosed with type 1 diabetes or if positive for GAD65 antibodies during treatment and follow-up. Although this study is cross-sectional, diabetes classifications were applied retrospectively to ensure that the population under study had type 2 diabetes. The study period was either 3 months before, or up to 12 months after, the date of diagnosis. All anthropometric measures and biochemical test results are limited to the study period. If there were multiple measures in that time, the measure closest to the date of diagnosis was used. Analyses were restricted to individuals diagnosed over the age of 18 years. Further details on cohorts are provided in ESM Methods: Cohort Information.

### Clinical data

Clinical variables were analysed only from the INSPIRED DMDSC cohort. Variables included age of diagnosis, sex, HbA_1c_, BMI, systolic and diastolic blood pressure (SBP and DBP), total cholesterol, HDL-cholesterol (HDL-C), LDL-cholesterol (LDL-C), triacylglycerol, serum creatinine and liver enzymes. These were measured in the fasted state. LDL-C was calculated using the Friedewald formula. Information such as family history of diabetes in first-degree relatives, fasting and postprandial C-peptide, HOMA-B and HOMA of insulin sensitivity (HOMA-S) were available. HOMA-B and HOMA-S were calculated from fasting C-peptide and glucose using the HOMA calculator [29–31].

### Genetic data: pPS for insulin secretion

pPS for type 2 diabetes risk have been developed [32, 33]. We used the beta cell dysfunction cluster consisting of eight variants classified as ‘Insulin secretion 1’, which is aetiologically distinct from other causal clusters. The distribution of the unweighted pPS was examined. A greater number of risk alleles implies increased risk for type 2 diabetes due to poor beta cell function. To compare the genetically determined differences in beta cell function, the variants were weighted for their association with a surrogate estimate of beta cell function (HOMA-B) [33]. A lower weighted pPS implies lower genetically determined beta cell function. A subgroup of Asian Indians who had been genotyped and met clinical study inclusion criteria (*n* = 1513) were used to examine the relationship between the pPS, age at diagnosis and BMI. Replication in the UK Biobank was undertaken using individuals diagnosed with type 2 diabetes [34]. We compared the distribution of the pPS risk variants in white British and South Asians populations. Ethnicity in UK Biobank was classified using self-reported ethnicity cross-validated with principal components analyses [35]. Information on genetic data quality control measures is provided in ESM Methods: Genotype Data Quality.

### Study design

Cross-sectional analyses were undertaken using the timepoint of diabetes diagnosis in all cohorts. Young-onset diabetes was defined as diagnosis at or under the age of 40 years [36]. Sensitivity analyses using population-derived age cut-offs were performed. The use of ethnicity-specific BMI cut-offs to define normal, overweight and obesity is recommended [37, 38]. We utilised a consensus definition in Asian Indians recommending thresholds of <23 kg/m^2^ and >25 kg/m^2^ as normal BMI and obesity [39, 40]. Normal BMI in white Europeans was defined as <25 kg/m^2^, and obesity was defined as BMI >30 kg/m^2^. We then examined risk factors for young lean-onset diabetes in Asian Indians in the DMDSC cohort. Finally, a comparison of the pPS for beta cell function in Asian Indians (DMDSC) and white Europeans (ESDC) with type 2 diabetes was undertaken. Details of the statistical methods used are provided in ESM Methods: Statistical Analyses.

### Ethics committee approvals

For the Indian cohort, ethics approval was granted by the National Institute for Health Research Global Health Research Unit on Global Diabetes Outcomes Research, Institutional Ethics Committee of Madras Diabetes Research Foundation, Chennai, India (IRB number IRB00002640, granted 24 August 2017). For the Scottish cohort, ethical approval for the study was provided by the Tayside Medical Ethics Committee (REF:053/04) The study was performed in accordance with the Declaration of Helsinki.

## Results

**Fig. 1:**
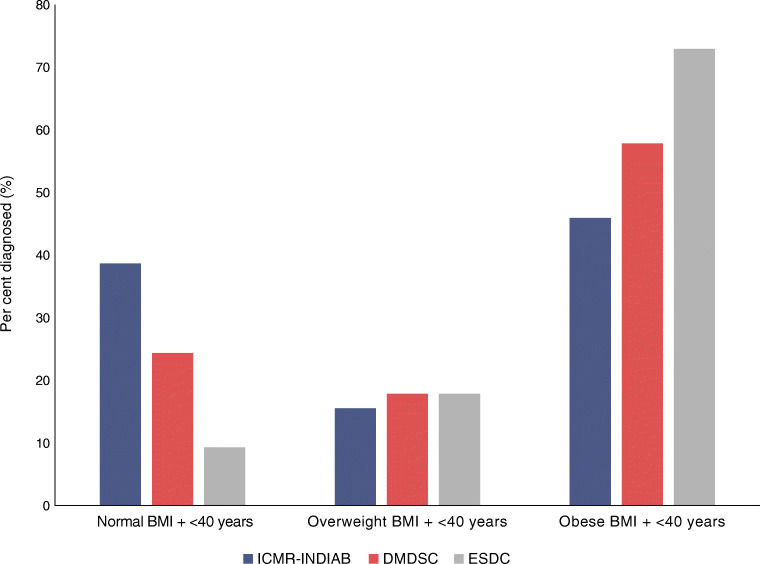
Bar graphs for Asian Indians (blue, ICMR-INDIAB cohort; red, DMDSC cohort) and white Europeans (grey, ESDC cohort) with early-onset diabetes by proportion belonging to each BMI category. The normal BMI for Asian Indians was <23 kg/m^2^; overweight was defined as BMI 23–25 kg/m^2^, obese was BMI >25 kg/m^2^. The normal BMI for the white European population was <25 kg/m^2^; overweight was defined as BMI 25–30 kg/m^2^, obese was BMI >30 kg/m^2^ [16]. Early onset for both ethnicities was defined as those diagnosed at 40 years or younger (<40 years). Further data on cohorts are available in ESM Fig. 1 and ESM Table 1

A total of 1712 Asian Indians from the ICMR-INDIAB cohort, 54,989 Asian Indians from the DMDSC cohort and 42,563 white Europeans from the ESDC, all with newly diagnosed type 2 diabetes, were categorised by their age at diagnosis and BMI (Fig. 1). Their characteristics are summarised in ESM Table 2. The median age of diagnosis amongst Asian Indians in the ICMR-INDIAB cohort was 50 years, that in the DMDSC cohort was 47 years, and that in white Europeans was 62 years (ESM Fig. 1).

### Early-onset diabetes with normal BMI occurs at over twice the frequency in Asian Indians compared with white Europeans

Ethnicity-specific BMI cut-offs for normal, overweight and obesity were applied. As seen in Fig. 1, the majority of Asian Indians who were diagnosed young were obese on the basis of their BMI (>25 kg/m^2^): 45.9% in the ICMR-INDIAB cohort and 57.8% in the DMDSC cohort. Similarly, 72.9% of white Europeans were obese on the basis of their BMI (>30 kg/m^2^). However, a shift of distribution was observed for young-onset diabetes with normal BMI: 39% of Indians from the ICMR-INDIAB study and 24% of Indians from the DMDSC cohort were classified as having normal BMI (<23 kg/m^2^), in contrast with 9.3% of white Europeans (<25 kg/m^2^) (χ^2^ = 25, *p*<0.0001). A sensitivity analysis using population-derived age cut-offs showed similar results (χ^2^ = 9.8, *p*=0.001) (ESM Fig. 2).

### Asian Indians diagnosed young have lower beta cell function compared with those diagnosed at an older age

Individuals from the DMDSC cohort were divided into two age groups: young diagnosed (individuals with age of diagnosis of 40 years or younger) and older diagnosed (>40 years). The distribution of clinical and anthropometric features across age groups is described in Table 1. Both fasted and stimulated C-peptide were significantly lower in those diagnosed young compared with those diagnosed at an older age. Beta cell function, determined using HOMA-B, along with fasted and stimulated C-peptide levels, were also lower in young compared with older diagnosed individuals. While C-peptide levels were measured in a small subgroup of patients, their absence was comparable across BMI categories (86%, 84% and 84% in normal, overweight and obese groups). A family history of diabetes was observed more frequently in younger individuals. Amongst those diagnosed young, a higher proportion were male (71%), despite more male participants having normal/healthy waist circumferences (54%) than female participants (39%). No difference in mean BMI was observed between the two groups. Comparative dyslipidaemia, characterised by higher triacylglycerol and lower HDL-C, was observed in young individuals compared with older individuals; however, LDL-C levels were lower in young individuals. The levels of the liver enzyme alanine aminotransferase (ALT) were significantly higher in younger diagnosed individuals. Overall, a pattern of poor beta cell function along with increased adiposity and adverse lipid and liver profiles was observed in young Asian Indians with diabetes. These features were then examined across BMI categories amongst those diagnosed young (ESM Tables 3 and 4).

**Table 1:** Comparison of characteristics between young and older diagnosed Asian Indians

Clinical characteristics	Young diagnosed (≤40 years)		Older diagnosed (>40 years)	
	*n*		*n*	
Age at diagnosis (years)	15,263	34.1 (31.2–37.7)	39,726	52.6 (45.7–57.8)
Sex (%)				
Female*	4407	29	11,953	41
Male	10,856	71	16,862	59
BMI (kg/m^2^)	14,314	26.7 (23.7–29.1)	37,526	26.7(23.7–29.1)
Waist circumference (cm)^a^				
Female*	3141	90.4 (83–97)	11,953	91.0 (84–98)
Male*	7947	93.5 (87–99)	16,862	95.2 (88–101)
Healthy waist circumference (%)^a^				
Female*	1714	39	5744	35
Male*	5846	54	11,432	49
HbA_1c_ (%)*	15,263	8.8 (7.2–10.7)	39,726	8.2 (6.9–10.2)
HbA_1c_ (mmol/mol)*	15,263	72.6 (55.2–93.4)	39,726	66.1 (51.9–88.0)
Fasted C-peptide (pmol/ml)*^,b^	3183	1000 (700–1300)	5510	1000 (800–1300)
Stimulated C-peptide (pmol/ml)*^,b^	3183	2100 (1500–3000)	5510	2600 (1900–3500)
Stimulated C-peptide adjusted for insulin sensitivity*^,b^	3183	606 (406–891)	5510	730 (519–1008)
HOMA-S *^,b^	3183	35.4 (27.0–47.6)	5510	35.1 (26.9–45.2)
HOMA-B *^,b^	3183	47.5 (26.3–83.2)	5510	68.5 (36.9–108.3)
Family history of diabetes (% with family history)*	14,866	69	38,583	44
LDL-C (mmol/l)*	13,116	2.95 (2.38–3.54)	33,818	3.04 (2.40–3.62)
HDL-C (mmol/l)*	13,116	0.96 (0.85–1.11)	33,818	1.06 (0.91–1.19)
Triacylglycerol (mmol/l)*^,b^	13,116	1.76 (1.26–2.60)	33,818	1.63 (1.21–2.25)
Total cholesterol (mmol/l)	13,116	4.88 (4.22–5.61)	33,818	4.94 (4.24–5.66)
ALT (U/l)*^,b^	7410	32 (21–48)	18,559	24 (18–35)

Data are mean (SD), median (IQR) or %

^a^The healthy waist circumference guideline for Asian Indian men is 90 cm and that for women is 80 cm

^b^Statistical tests were performed on log-transformed variables

*Differences were statistically significant (*p*<0.05)

### Asian Indians diagnosed young with normal BMI have lower beta cell function compared with those diagnosed young who are overweight or obese

**Fig. 2:**
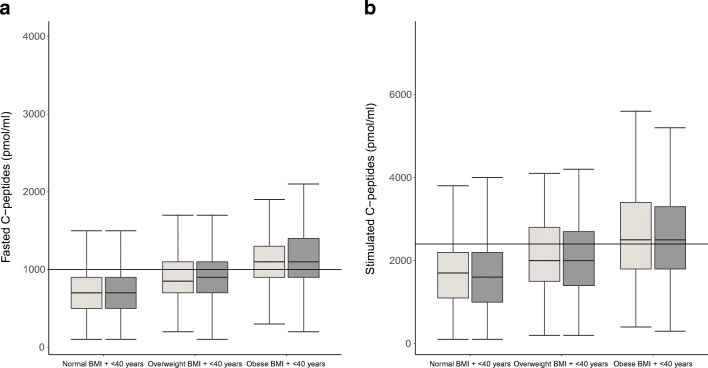
Boxplots demonstrating lower fasted C-peptide levels (**a**) and stimulated C-peptide levels (**b**) by BMI category. Data shown are from the DMDSC cohort of Asian Indians with type 2 diabetes. The normal BMI for Asian Indians was <23 kg/m^2^; overweight was defined as BMI 23–25 kg/m^2^, obese was BMI >25 kg/m^2^. Early onset is defined as those diagnosed at 40 years or younger (< 40 years). Light grey, male participants; dark grey, female participants. Those with normal BMI compared with those classified as overweight and obese had lower fasted and stimulated C-peptide levels and also lower beta cell function, with a compensatory increase in insulin sensitivity. When adjusted for insulin sensitivity, the association between stimulated C-peptide levels and age at diagnosis remained significant (*p*<0.0001)

An examination of trends across BMI categories in the two age groups is provided in ESM Table 3. In young diagnosed individuals, those with normal/lean BMI had lower fasted and stimulated C-peptide levels compared with overweight and obese individuals (Fig. 2). This effect persisted when stimulated C-peptide levels were adjusted for insulin sensitivity (HOMA-S). Similarly, HOMA-B was lower in the lean individuals compared with the overweight and obese groups, while HOMA-S was higher (ESM Fig. 3). Young diagnosed individuals with normal BMI had lower triacylglycerols and higher HDL-C compared with those who were diagnosed young and were overweight or obese (ESM Fig. 4). Together, these results suggest that diabetes in young lean Asian Indians is not selectively associated with dyslipidaemia, but more likely with impaired or poor beta cell function.

The observation of impaired beta cell function in young lean Asian Indians persisted after adjusting for sex, family history of diabetes, HDL-C and insulin sensitivity (ESM Table 4). The adjusted difference in stimulated C-peptide levels between those diagnosed with normal BMI compared with those who were obese was 492 pmol/ml (IQR 353–616). The adjusted difference in untransformed HOMA-B was 25.16 (IQR 20.6–28.6).

### The pPS for type 2 diabetes risk due to poor beta cell function is lower in Asian Indians compared with white Europeans

**Fig. 3:**
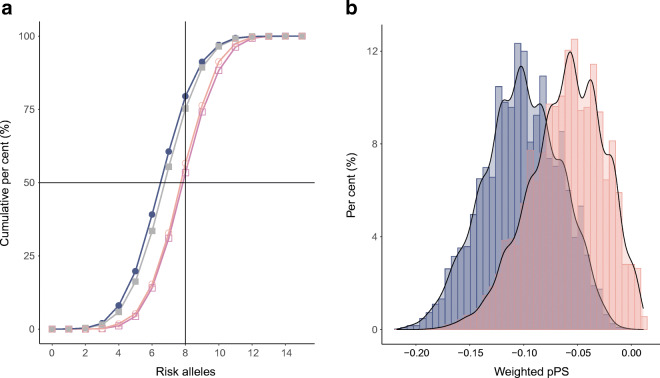
(**a**) Cumulative distribution of risk alleles for beta cell function pPS in all populations. The closed shapes are white Europeans and open shapes are South Asians. Dark pink open squares are Asian Indians from INSPIRED DMDSC cohort; light pink open circles are South Asians from UK Biobank; blue closed circles are white Europeans from INSPIRED ESDC, grey closed squares are white Europeans from UK Biobank. The difference in distribution of risk alleles (unweighted pPS) was significantly different between ethnicities in both study cohorts (Wilcoxon–Mann–Whitney *p*<0.0001). (**b**) Comparative histogram showing differences in the distribution of pPS weighted for HOMA-B in INSPIRED cohorts. Blue, white Europeans from INSPIRED ESDC; pink, Asian Indians from the INSPIRED DMDSC cohort. The difference in distribution was significantly different between ethnicities (Wilcoxon–Mann–Whitney *p*<0.0001)

In total, 5806 Asian Indians and 6933 white Europeans with type 2 diabetes from the INSPIRED study cohorts were included in this analysis. The replication from UK Biobank included 1153 South Asians and 17,183 white Europeans with type 2 diabetes. We used the ‘insulin secretion’ pPS of variants in eight genes associated with type 2 diabetes risk due to poor beta cell function: *MTNR1B*, *HNF1A*, *GCK*, *TCF7L2*, *TMEM258*, *ADCY5*, *SLC3OA8* and *ABO* [32, 33]. Seven out of eight variants used in the pPS were directly typed in our cohorts. Risk alleles across these variants were summed for the INSPIRED cohorts and UK Biobank, allowing the comparison of an unweighted partitioned risk score (pPS) across ethnicities (ESM Table 5 and ESM Fig. 5). The pooled distributions of these risk alleles across the INSPIRED and UK Biobank cohorts are provided in Fig. 3a. In both the INSPIRED and UK Biobank cohorts, Asian Indians and South Asians had a higher number of risk alleles compared with white Europeans (*p*<0.0001). The approximate percentage of white Europeans with eight or more risk alleles was 25%, whereas that for South Asians or Asian Indians was 45%. The difference in the empirical distribution of risk alleles between the two ethnicities was significant (Kolmogrov–Smirnov *p* value <0.0001) (ESM Fig. 6).

In the INSPIRED cohorts, risk alleles were weighted for their association with HOMA-B (Fig. 3b) to capture genetically determined beta cell function associated with these variants. A lower weighted pPS corresponds to lower HOMA-B and beta cell function. The weighted pPS was found to be non-parametrically distributed in both populations (ESM Table 6). A comparison showed that Asian Indians have lower genetically determined beta cell function compared with white Europeans (*p*<0.0001) (Fig. 3b). Higher weighted pPS was strongly associated with increasing age of diagnosis in Asian Indians (*Z* = 5.2, *p*<0.0001) (ESM Fig. 7), but no trend or association was observed in white Europeans (*Z* = 1.1, *p*=0.15) (ESM Fig. 7). In Asian Indians, the trend for the pPS across age groups mirrored that of stimulated C-peptide levels, HOMA-B and beta cell function adjusted for insulin sensitivity (ESM Fig. 8), with those with onset at younger ages having lower stimulated C-peptide levels, HOMA-B and genetically determined beta cell function than individuals with older onset.

A subgroup of the genotyped Asian Indian cohort who met the clinical study inclusion criteria, i.e. for whom BMI was measured at diagnosis of type 2 diabetes, was available (*n* = 1513). We used linear regression models stratified by BMI to predict age at diagnosis using the pPS in Asian Indians. We observed that the maximum variance explained was in those with normal BMI (*R*^2^ = 1.2%), with decreasing variance for overweight individuals (*R*^2^ = 0.97%) and obese individuals (*R*^2^ = 0.36%) (ESM Fig. 9 and ESM Table 7).

## Discussion

### Summary of key results

As far as we are aware, this study is the first to use non-migrant populations and ethnicity-specific BMI thresholds to highlight that the age of diabetes onset in Asian Indians is dramatically lower than in white Europeans, and that young onset is characterised by a two to four times higher prevalence of individuals with normal BMI. Further, we show that, amongst Asian Indians, this group (normal BMI + young onset) has a lower beta cell function. Using pPS values, we demonstrate that Asian Indians have a higher genetic burden of risk variants for lower beta cell function. Finally, we demonstrate that the pPS predicts age of diabetes diagnosis in those with normal BMI (*R*^2^ = 1.2%) better than in those classified as overweight or obese.

Although this study is focused on Asian Indians, many studies have used less specific South Asian populations, which may include people from Afghanistan, Pakistan, India, Bangladesh, Nepal, Bhutan and Sri Lanka etc. This is an important distinction, as genetic studies have shown significant heterogeneity in these populations that may be reflected in different clinical phenotypes [41, 42]. Additionally, thresholds for adiposity are considered to be different in these ethnic subgroups [39]. This, in addition to the fact that we are studying non-migrant populations, should be considered when comparing findings. In the UK Biobank replication study, the South Asian cohort (*n* = 1153) predominantly comprises individuals of Asian Indian descent (*n* = 700). In contrast, the white population in UK Biobank comprises a mixture of individuals of English, Welsh, Irish and Scottish origin, as well as of other European descent living in the UK. In contrast, the white Scottish population in the ESDC is from the local population of Tayside, Scotland.

We used two methods to determine young onset: a single threshold of young-onset diabetes (<40 years) and ethnicity-determined mean age of diagnosis (Fig. 1 and ESM Fig. 2). Using either approach, our results show that there is a preponderance of younger-onset diabetes in Asian Indians with a normal or healthy BMI. This difference is particularly striking as the mean age of diabetes onset is 12 years lower in Asian Indians, demonstrating that diabetes occurs earlier and in leaner individuals in this ethnic group. It is possible that the lower mean age of the underlying Indian compared with the Scottish population may be driving the differences in age at diagnosis. However, as these differences have also been documented in migrant Asian Indian and South Asian populations, it is likely that population structure alone is not driving the observed difference in age at diagnosis.

The phenomenon of lean, young onset of diabetes is particularly pronounced amongst Asian Indian men, who are more likely than women to have healthy waist circumferences when diagnosed young. There is no clear evidence of dyslipidaemia in the young-onset normal weight group compared with those with higher BMI. Given the reciprocal relationship between beta cell function and insulin sensitivity, it is not surprising that the insulin sensitivity based on the surrogate estimate of HOMA-S was higher in the lean young group compared with the obese young group. The genetic studies establish that it is the beta cell deficiency that is causal, with the increased HOMA-S secondary to this.

It is worth noting that the substantive proportion of young-onset diabetes in Indians occurred in those classified as obese on the basis of BMI (54% in the ICMR-INDIAB cohort and 57% in the DMDSC cohort); this group also has lower insulin sensitivity and higher ALT levels. This is suggestive of higher ectopic fat deposition, as ALT is a reasonable correlate of ectopic fat, consistent with the hypothesis of the ‘thin–fat’ phenotype that has been suggested to predispose Asian Indians to type 2 diabetes [9, 23, 43]. This mirrors the more classic insulin resistance aetiology of diabetes.

Our results suggest that the preponderance of young and lean Asian Indians developing diabetes is driven by poor beta cell function. The demographics of India are changing, with more urbanisation and a shift to sedentary jobs [44]. It is likely that, against the background of poor beta cell function and lower muscle mass, smaller increases in ectopic fat and adiposity may drastically increase diabetes risk. Furthermore, the trend with family history, which is strongly associated with obesity in our data, may be suggestive of a shared genetic risk for obesity, dyslipidaemia etc., but may also be reflective of shared lifestyle. Addressing the diabetes epidemic in India, and South Asia more broadly, will require efforts in diet awareness and exercise.

### Limitations

As yet, there has been no conclusive genetic study of beta cell function, HDL-C, obesity, lipodystrophy etc. in individuals of Asian Indian or, more broadly, South Asian ancestry. This prevents us from utilising an ethnicity-specific polygenic risk score. It is possible that such a study would produce a different or expanded set of candidate SNPs for beta cell function, as the pPS used in this study has been derived from studies in white European populations. Furthermore, studies on causal genetic variants of beta cell function are not available. Instead, the SNPs used have been shown to be statistically associated with diabetes risk due to poor beta cell function [32]. In our study, data were available for both C-peptide levels and genetic variables for 322 Asian Indians. We observe that both the unweighted and weighted pPS were correlated with adjusted stimulated C-peptide levels (*R*_unweighted_ = 12%, *p*=0.04; *R*_weighted_ = 14%, *p*=0.01). Notably, in the discovery study (INSPIRED DMDSC and ESDC), these SNPs had no pleiotropic effects on other glycaemic traits or diabetes risk factors, making them powerful genetic instruments. If these SNPs are not causal variants, they are tagging the causal variants through high linkage disequilibrium. It is possible that the linkage disequilibrium structure in these regions may differ between individuals of European and Asian ancestry. However, a recent trans-ancestral study of glycaemic traits has replicated many of these signals and reported that, overall, 80% of SNPs identified in single-ancestry studies of glycaemic traits showed no evidence of between-ancestry heterogeneity [45]. The association of the pPS with age of diagnosis in Asian Indians (ESM Fig. 7), mimicking the trend of stimulated C-peptide and HOMA-B (ESM Fig. 8), demonstrates its validity in this non-European cohort. In our data, the allele frequencies of the variants show differences across the two ethnicities (ESM Table 4 and ESM Fig. 5). We observed that, of the eight variants, Asian Indians had a higher risk allele frequency for six. This difference in burden of risk alleles was replicated in the UK Biobank data.

Due to the lack of routine screening for diabetes in the Indian population, HbA_1c_ at diagnosis is high. It is likely that the true age of onset is lower than the age recorded. However, this differential bias is likely to underestimate our finding of early age of diabetes onset with lean BMI.

Another limitation is the lack of comparison of glycaemic traits between the two ethnicities, particularly C-peptide levels. However, we do not expect to see a different relationship between BMI (as a measure of adiposity) and glycaemic traits in white Europeans than in Asian Indians, i.e. diabetes onset with low BMI is likely to be associated with beta cell failure [46]. Our conclusion, supported by the comparative genetic study, is that lower beta cell function is observed more frequently in Asian Indians with type 2 diabetes. Finally, a recurring hypothesis in the aetiology of type 2 diabetes in South Asians involves low lean or muscle mass [21, 23]. Indeed, studies have found that South Asians have low grip strength compared with white Britons [47]. Unfortunately, we do not have the data to examine this. However, this is a key area of future research.

### Strengths

The availability of an intensively phenotyped cohort of non-migrant Asian Indians is novel, and inclusion of data on glycaemic traits, such as fasted and stimulated C-peptide levels, anthropometric traits such as BMI and waist circumference, lipid traits and family history, is unique. Availability of genetic data is an added advantage. The comparative distribution of the pPS between white Europeans and Asian Indians demonstrates an unbiased estimate of the genetic burden of lower beta cell function in Asian Indians. Furthermore, the observed difference in burden of risk alleles was replicated using the UK Biobank, comparing broader ethnic group of South Asians with white British individuals. The results are consistent with the prevailing hypothesis that beta cell function is lower in Asian Indians and more broadly in South Asian populations [21, 23], which predisposes them to diabetes even at low BMI.

### Generalisability

The data were obtained from a private healthcare centre in India, and are more likely to reflect the situation in middle- and upper middle-class residents of urban India. However, no evidence exists suggesting that the underlying genetic architecture in urban and rural India is different. Therefore, we expect that the phenotype of diabetes driven by lower beta cell function will persist in rural areas. Indeed, this is clearly evidenced by the high rates of impaired fasting glucose prevalence observed in these settings [2]. Moreover, we also included data from the ICMR-INDIAB cohort, which is a national survey of diabetes and prediabetes prevalence in a representative population of India, and found a nearly equivalent age-adjusted prevalence of impaired fasting glucose in urban and rural-dwelling Indians of approximately 10% [2].

### Interpretation

These results show that lower clinically and genetically determined beta cell function contributes to the higher burden of younger-onset type 2 diabetes in Asian Indians. Our results are supported by clinical clustering studies showing that, in contrast to white Europeans, Asian Indians with young-onset diabetes have a preponderance of insulin deficiency, suggesting a role of beta cell dysfunction [48, 48].

## Supplementary information

ESM 1 (PDF 702 KB)

## Data Availability

Data used in this study are restricted as they are obtained from unconsented population and clinical cohorts, and are only analysable in secure computing environments hosted by the University of Dundee and the Madras Diabetes Research Foundation. Requests for data access may be made directly to the corresponding authors.

## Abbreviations

ALT: Alanine aminotransferase
DMDSC: Dr Mohan’s Diabetes Specialties Centre
ESDC: East Scotland Diabetes Cohort
HDL-C: HDL-cholesterol
HOMA-S: HOMA of insulin sensitivity
ICMR-INDIAB: Indian Council of Medical Research-India Diabetes Study
INSPIRED: India–Scotland Partnership for Precision Medicine in Diabetes
LDL-C: LDL-cholesterol
pPS: Partitioned polygenic score
