# Correction to: Young-onset diabetes in Asian Indians is associated with lower measured and genetically determined beta cell function

**DOI:** 10.1007/s00125-022-05707-4

**Published:** 2022-04-26

**Authors:** Moneeza K. Siddiqui, Ranjit Mohan Anjana, Adem Y. Dawed, Cyrielle Martoeau, Sundararajan Srinivasan, Jebarani Saravanan, Sathish K. Madanagopal, Alasdair Taylor, Samira Bell, Abirami Veluchamy, Rajendra Pradeepa, Naveed Sattar, Radha Venkatesan, Colin N. A. Palmer, Ewan R. Pearson, Viswanathan Mohan

**Affiliations:** 1 National Institute for Health Research Global Health Unit for Diabetes Outcomes Research, Division of Population Health & Genomics, School of Medicine, University of Dundee, Dundee, UK; 2 Dr Mohan’s Diabetes Specialities Centre and Madras Diabetes Research Foundation, Chennai, India; 3 Institute of Cardiovascular and Medical Sciences, University of Glasgow, Glasgow, UK

**Correction to: Diabetologia**

10.1007/s00125-022-05671-z

The following sentences have been removed from the supplementary methods as no comparisons were made with white European participants from the ADOPT trial and the UKPDS trial: ‘Comparison was made with white European participants from the ADOPT trial [4] and the UKPDS trial [5, 6]. Since different assays have been used to measure fasting glucose and insulin or c-peptide in these populations the HOMA results have been plotted separately to highlight the difference in overall trend, not the absolute difference in levels’. The original ESM has been corrected.

