# Supplementary material for: Young-onset diabetes in Asian Indians is associated with lower measured and genetically determined beta cell function
Source: Diabetologia. 2022 Mar 5;65(6):973–83. doi: 10.1007/s00125-022-05671-z (PMC9076730; doi:10.1007/s00125-022-05671-z)
Supplement: Supplementary file 1 — (PDF 702 KB) [file 125_2022_5671_MOESM1_ESM.pdf]

Electronic supplementary materials

ESM: Methods

1. Supplemental Cohort information

INSPIRED-East Scotland Diabetes Cohort (ESDC)

In Scotland, all clinical data for patients with diabetes is captured on an electronic medical record system: Scottish Care Information - Diabetes Collaboration (SCI-Diabetes). As all patients with diabetes are managed within the National Health Service, there is complete capture of patient data for all patients from diagnosis. The East of Scotland (Tayside and Fife, population 800,000) has approximately 15 years of complete population capture of prescription encashment. The population included in this study was therefore patients diagnosed with type 2 diabetes after 1994 from Tayside and Fife, who met all other study criteria.

Controls (individuals who did not have diagnosis of type 2 diabetes) were sourced from the GoDARTS study [1] where 8157 controls were recruited between October 2004 and May 2009. Anthropometric lifestyle measurements were recorded for this group at the time of recruitment. Additionally, all patients attend a clinic at recruitment where biochemistry tests are performed, this is the source of the HDL data reported.

All electronic health records were anonymised and made available through the Health Informatics Centre (HIC) in Dundee, with all analysis undertaken on the ISO270001 approved HIC data safe haven.

INSPIRED- INDIAB: Asian Indian national cohort

The ICMR-INDIAB study was performed using a multi-stage reporting cross-sectional [2]. Data used for this analysis are from the first two phases and the north east phase of this population-survey, performed between November 2008 and July 2015 [3]. Anthropometric and clinical data was surveyed from representative participants belonging to 15 states in India. Capillary oral glucose tolerance tests were used to diagnose diabetes per WHO criteria.

As part of this survey to estimate the prevalence of diabetes and pre-diabetes a variety of anthropometric, clinical, socio-economic and lifestyle factors were recorded. We report findings data from 14 Indian states (out of 28) and 1 union territory.

INSPIRED - DMDSC: Asian Indian diabetes-specialty cohort

Dr Mohan’s Diabetes Speciality Centre (DMDSC) is a privately-run chain of single speciality hospitals and clinics for treatment of diabetes and associated disorders. The

institution has been in operation since 1991 and currently there are 50 clinics in various locations across 10 states in India, all connected through a single electronic medical system. At present, there are approximately 500,000 patients with Type 2 diabetes within DMDSC, who comprise our Asian Indian cohort; 55,148 whose first clinic visit was within 1 year of diagnosis are presented here.

Patients registered any of the clinics are given a unique ID which is linked to the common Diabetes Electronic Medical Records. All investigations done including biochemical testing, imaging, anthropometric measurements, retinal exams, doppler, biothesiometry or surgical treatments can be viewed by physicians across clinics giving the patient freedom to access any clinic of his/her choice. All test results are directly fed into the system. Physician interaction notes and details of prescriptions issued are available in the system. Pharmacy records are also fed into the system with all the above linked to the unique patient ID. The Madras Diabetes Research Foundation (MDRF) is the research wing of DMDSC and helps in data management and mining. The DMDSC laboratory is accredited by College of American Pathologists (CAP) as well as by the National Accreditation Board for Testing and Calibration Laboratories (NABL) for measurement of glucose, insulin and c-peptide.

Clinical variables

HOMA in the DMDSC cohort was derived using fasting c-peptide in patients who were diet treated, treated with oral antihyperglycaemics, and in a small percentage, with insulin.

ESM Table 1. Data contribution by study cohort

Data contributed\Cohort name	INDIAB (pan-India)	INSPIRED-ESDC (East Scotland, UK)	INSPIRED-DMDSC (Chennai, India)	UK Biobank
BMI and age of diagnosis	✓	✓	✓	
Clinical measures			✓	
Partitioned polygenic scores		✓	✓	✓

ESM Methods

2. Genotype data quality

Genetic data from the white European ESDC cohort was available from four platforms: Affymetrix 6.0 Affymetrix, Santa Clara), Illumina OmniExpress -12VI platform and Illumina Infinium (Illumina, San Diego) for a total of 6,933 white Europeans with

diabetes [1]. Genetic data for DMDSC was available from the Illumina Global Screening Array for 5,806 Asian Indians with type 2 diabetes. Post genotyping quality-control procedures were applied to each dataset separately. Imputation was performed against the Haplotype Reference Consortium (HRC) panel separately for both populations [4] using the Michigan Imputation Server and Sanger Imputation Server. HRC contains data from 1000G which incorporates data from South Asian genomes. All variants had a genotype call rate > 90% and quality threshold of 0.90 was applied for imputed SNPs. All genotyped individuals had a confirmed diagnosis of type 2 diabetes per criteria described above. All variants considered were in Hardy-Weinberg equilibrium in their respective populations (p value $<10^{-4}$).

ESM Methods

3. Statistical analyses

The distributions of clinical and anthropometric traits as well as the pPS were assessed for normality. Means and standard deviations were computed for normally distributed variables and medians and inter-quartile ranges were computed for all non-parametric variables. Tests for trend across age and BMI groups was performed. Test for trend for parametric variables were performed using linear regression (T statistic), non-parametric using Jonckheere-Terpstra test (Z statistic) and for categorical variables using Cochran-Armitage Trend test (Z statistic). Quantile regression analyses were undertaken to detect differences in glycemic traits across BMI groups in models adjusted for sex, family history, HDL-cholesterol, and insulin sensitivity. Finally, the comparative distribution of the pPS for beta cell function between Asian Indians and white Europeans with type 2 diabetes was assessed using the Wilcoxon-Mann-Whitney test for differences in median and Kolmogorov-Smirnov statistic for testing two-sample non-parametric distributions. Similarly, a replication in the UK Biobank comparing the distribution of the pPS in white British and South Asians with type 2 diabetes was performed. Jonckheere-Terpstra Z statistics were used to assess in the relationship between the pPS and age groups at diagnosis. Generalised linear models were used to test the association of the pPS with age of diagnosis. Statistical analyses and graphs were produced using SAS 9.4 (SAS Institute, Cary, North Carolina). Values are reported in SI units throughout.

ESM Results

1. Distribution of age of diagnosis and BMI categories

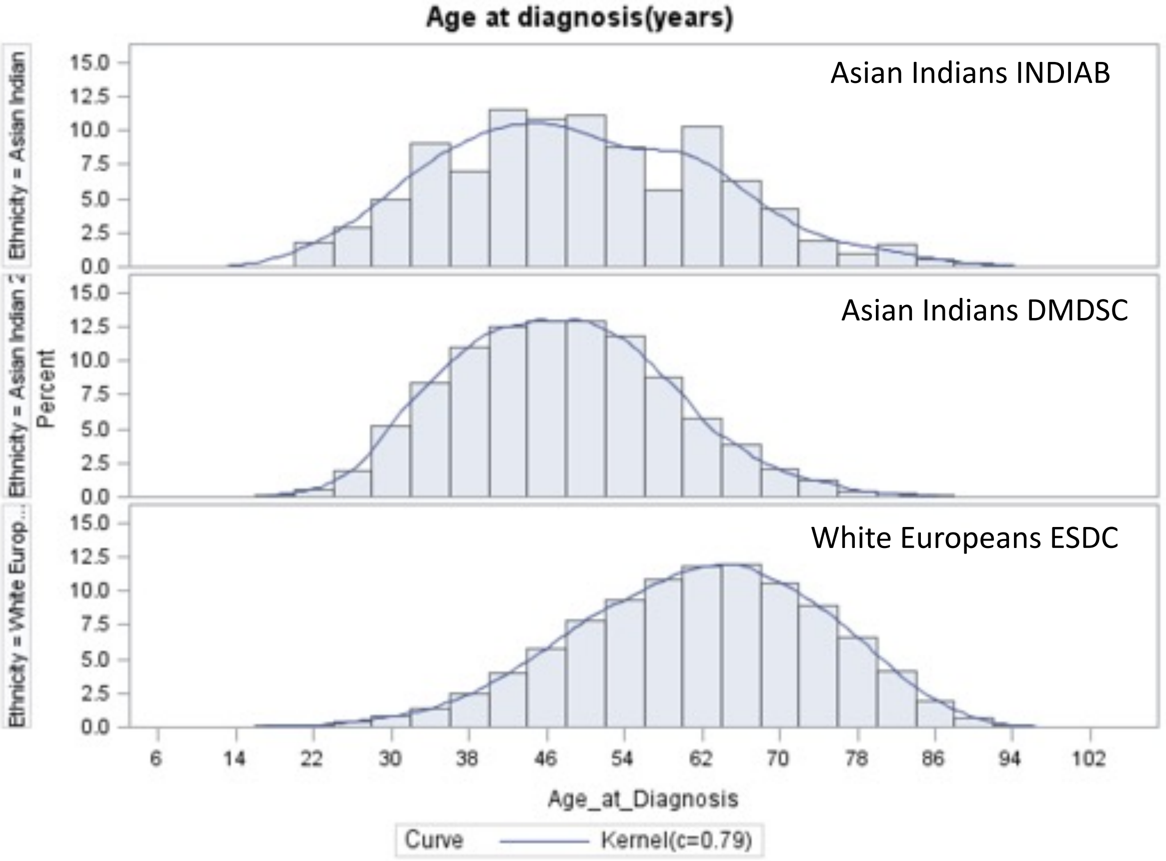

ESM Figure 1. Histogram of age of diabetes onset in Asian Indians from population sample - INDIAB (top panel), Asian Indians from a diabetes specialty clinic - DMDSC (middle panel) and white Europeans - ESDC (bottom panel). Median age of diagnosis for Asian Indians in INDIAB was 48 years, and in DMDSC was 47 years, while for white Europeans was 62 years.

ESM Table 2. Demographic characteristics of populations described in histograms

**Trait\Population**	**White Europeans (ESDC) (n= 42,563)**		**Asian Indian (INDIAB) (n = 1712)**		**Asian Indians (DMDSC) (n=54,989)**	
	Total n	*Mean (SD)*	*Total n*	*Mean (SD)*	*Total n*	*Mean (SD)*
**Sex (Female)%**	42,563	45%	1,712	53%	54,989	37.6%
**Average age (mean, SD) (years)**	42,563	61.7 (13)	1,712	50 (13)	54,989	47.4 (11.2)
**Median age (median, IQR) (years)**	42,563	62 (18)	1,712	48 (21)	54,989	47 (16)
**BMI (mean, SD) (Kg/m^2)**	39,234	32.3 (6.6)	1712	24.2 (4.8)	51,840	26.7 (4.5)
**Waist circumference (cm) (mean, SD)**	8,046	106.1 (14)	1681	86.2 (13)	39,903	93.3 (10.6)

ESDC: East Scotland Diabetes Cohort, INDIAB: Indian-Diabetes cohort, DMDSC: Dr. Mohan's Diabetes Specialty Clinic

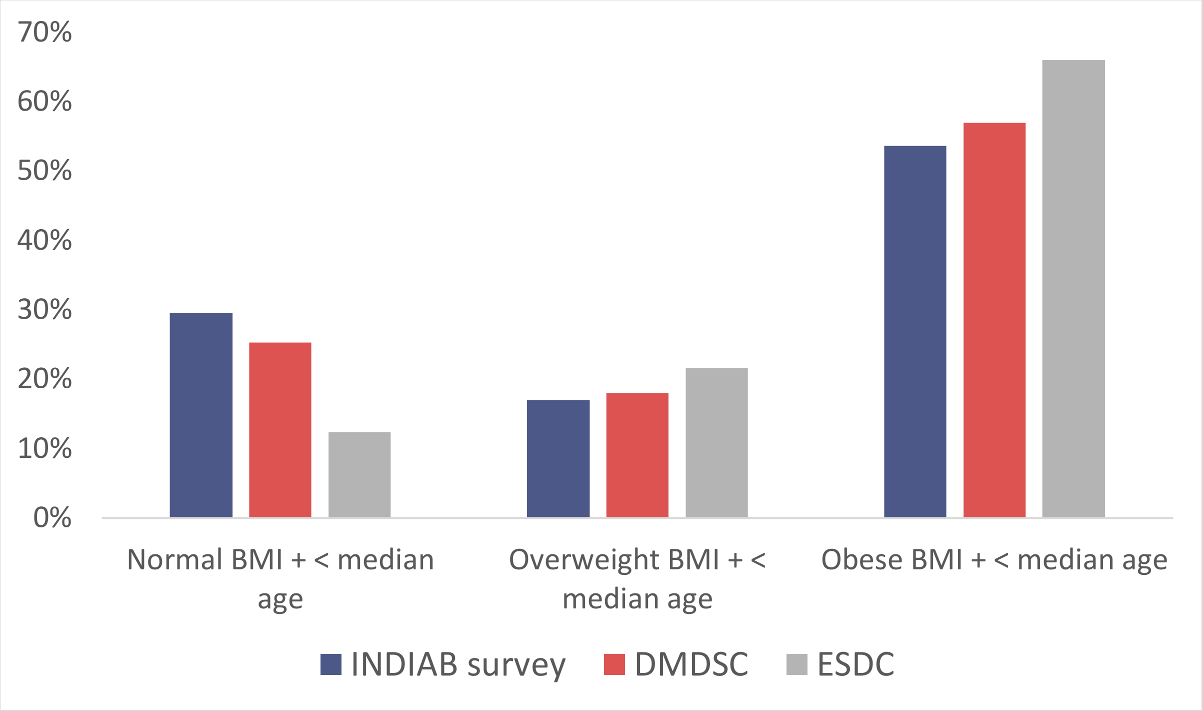

ESM Figure 2. Sensitivity analyses using population-derived age cut-offs. Bar graphs of Asian Indians (blue -INDIAB and red-DMDSC) and white Europeans (grey-ESDC) with early onset diabetes by proportion belonging to each BMI category. Normal BMI for Asian Indians was <23 kg/m2, overweight: 23-25kg/m2, obese: >25kg/m2. Normal BMI for white European population is <25 kg/m2, overweight 25-30 kg/m2 and obese >30 kg/m2 [16]. Since INDIAB and ESDC are a representative population, early or young onset diabetes in Asian Indians could also be defined as being under the age of 50 years, and under 62 for white Europeans.

2. Clinical features for young and older onset diabetes in Asian Indians: INSPIRED- DMDSC

ESM Table 3. Examining trends across BMI categories in young (≤40) and old (>40) diagnosed Asian Indians

	Young diagnosed (≤40 years)				Older diagnosed (>40 years)			
**Variables**	**Normal BMI**	**Overweight BMI**	**Obese BMI**	**Test for trend, P value**	**Normal BMI**	**Overweight BMI**	**Obese BMI**	**Test for Trend, P value**
**Numbers**	3716	2723	8824		9395	7012	23319	
**Age at diagnosis (years) (mean, SD)**	33.7 (4.5)	34.1 (4.3)	34.2 (4.3)	-2.03, 0.04	53.7 (9.4)	52.8 (8.7)	52.0 (8.1)	3.8, 0.0002
**Sex (% female)**	24%	25%	33%	10.9, <0.0001	33%	33%	46%	24.3, <0.0001
**BMI (kg/m^2) (mean, SD)**	21.2 (1.6)	24.1 (0.6)	29.3 (3.7)	--	21.1 (1.7)	24.1 (0.60)	29.2 (3.6)	--
**Waist circumference (females) (cm) (mean, SD)**	77.23 (7.7)	83.6 (6.5)	94.0 (9.6)	33.6, <0.0001	78.8 (7.4)	84.8 (6.4)	94.4 (9.3)	63.2, <0.0001
**Waist circumference (males) (cm) (mean, SD)**	81.9 (6.3)	88.3 (5.0)	98.9 (9.1)	67.6, <0.0001	84.0 (6.6)	90.6 (5.2)	100.7 (8.7)	98, <0.0001
**HbA1c (%) (mean, SD)**	9.7 (2.7)	9.3 (2.4)	8.9 (2.1)	5.6, <0.0001	9.12 (2.6)	8.84 (2.3)	8.56 (2.1)	3.6, 0.0004
**HbA1c (mmol/mol) (mean, SD)**	83 (30)	78 (26)	73.2 (24)	--	76 (29)	73 (25)	70 (23)	--
**Fasted c-peptide (pmol/mL) (median, IQR)**	700 (400)	900 (400)	1100 (500)	-9.7, <0.0001	800 (400)	1000 (500)	1100 (500)	-6.5, <0.0001
**Stimulated c-peptide (pmol/mL) (median, IQR)**	1600 (1200)	2000 (1400)	2500 (1600)	-5.4, <0.0001	2000 (1400)	2400 (1500)	2800 (1600)	-6.1, <0.0001

**Stimulated c-peptide adjusted for insulin sensitivity**	417 (333)	556 (392)	732 (481)	22.5, <0.0001	538 (366)	675 (469)	811 (473)	23.1, <0.0001
**HOMA-S (median, IQR)**	48.5 (27.8)	38.2 (20.7)	31.3 (15.0)	9.6, <0.0001	43.9 (25.2)	37.8 (19.1)	32.6 (15.6)	7.4, <0.0001
**HOMA-B (median, IQR)**	31 (41.7)	41.8 (44.2)	56.8 (61.3)	-4.6, <0.0001	50.2 (64)	61.4 (64.7)	76.1 (72.1)	-4.0, <0.0001
**Family history of diabetes (%)**	63%	67%	73%	-11.9, <0.0001	36.9%	43.0%	47.6%	-17.4, <0.0001
**LDL (mmol/L) (mean, SD)**	2.97 (0.94)	2.98 (0.90)	2.99 (0.90)	-1.6, NS	3.06 (0.96)	3.04 (0.94)	3.03 (0.92)	1.3, NS
**HDL (mmol/L) (mean, SD)**	1.03 (0.24)	1.00 (0.21)	0.98 (0.21)	-8.9, <0.0001	1.07 (0.26)	1.05 (0.24)	1.06 (0.24)	0.92, NS
**Triglycerides (mmol/L) (median, IQR)**	1.63 (1.30)	1.81 (1.39)	1.81 (1.33)	-4.9, <0.0001	1.57 (1.06)	1.67 (1.08)	1.63 (1.03)	-5.01, <0.0001
**Total Cholesterol (mmol/L) (mean, SD)**	4.94 (1.14)	5.00 (1.21)	4.94 (1.11)	-0.02, NS	5.01 (1.2)	4.99 (1.15)	4.96 (1.11)	-3.6, 0.0001
**ALT (IU/L) (median, IQR)**	26 (21)	31 (25)	34 (29)	-6.2, <0.0001	22 (14)	24 (16)	25 (17)	NS

Test for trend for parametric variables using linear regression (t statistic), non-parametric using Jonckheere-Terpstra test (Z statistic) and for categorical variables using Cochran-Armitage Trend test (Z statistic). Threshold for significance after correction for multiple testing = 0.001.

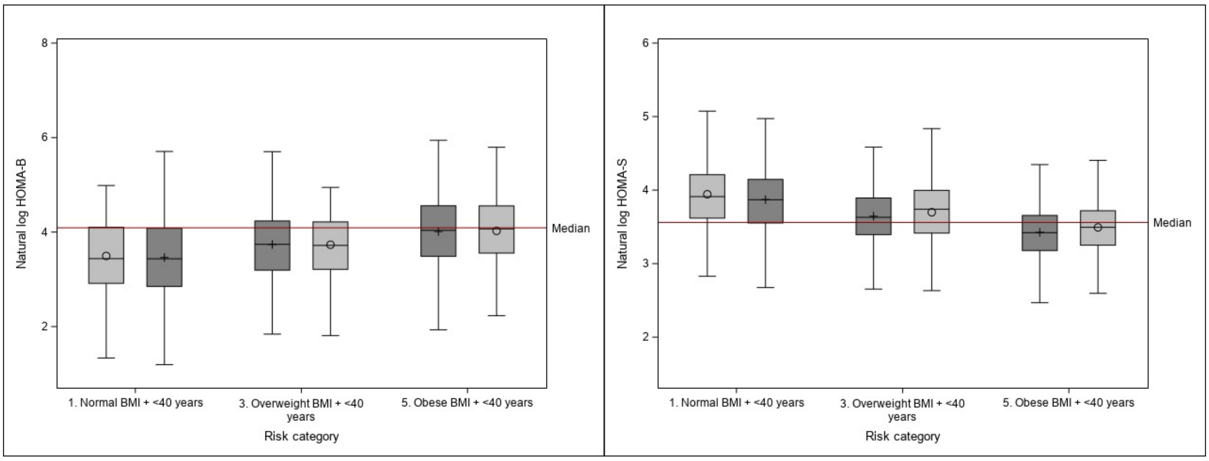

ESM Figure 3. Differences in HOMA-B (left) and HOMA-S (right) (both natural log transformed) in those diagnosed young across BMI categories. Males in light gray, females in dark gray. After adjusting for insulin sensitivity (HOMA-S) the association between BMI categories and HOMA-B remained significant (P<0.0001).

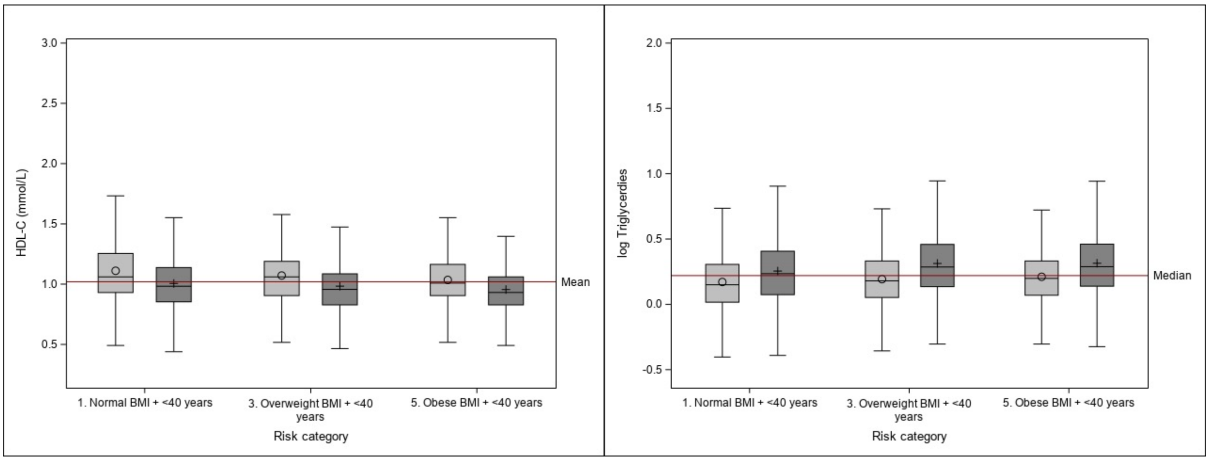

ESM Figure 4. Differences in HDL-C (left) and triglycerides (log transformed) in those diagnosed young across BMI categories. Males in light gray, females in dark gray.

ESM Table 4. Quantile regression models for glycemic traits showing adjusted differences across BMI groups in young diagnosed Asian Indians (diagnosis at age of 40 years or younger)

	**Median (and inter-quartile range) difference between successive BMI groups**	**Median (and inter-quartile range) difference between normal and obese groups**	**Test for trend across BMI categories**
**Fasting C-peptide levels (pmol/mL)**	167 (131, 202)	393 (362,424)	9.2, <0.0001
**Stimulated C-peptide levels (pmol/mL)**	371 (240, 503)	864 (763, 976)	5.5, <0.0001
**Stimulated C-peptide levels (pmol/mL) * adjusted for HOMA-S**	208 (77, 340)	492 (353, 616)	3.1, 0.002
**Differences in HOMA-S**	10.2 (7.6, 12.7)	16.97 (14.94, 18.66)	7.8, <0.0001
**Differences in HOMA-B**	9.9 (5.17, 14.7)	24.7 (20.2, 28.6)	4.08, <0.0001
**Differences in HOMA-B *adjusted for HOMA-S**	9.9 (5.12, 14.8)	25.16 (20.6, 28.6)	4.04, <0.0001

*Differences adjusted for sex, family history of type 2 diabetes, HDL-c levels. *Differences in HOMA-B and stimulated C-peptides were additionally adjusted for insulin sensitivity (HOMA-S). P value threshold after correction for multiple testing was 0.008.*

ESM Results 3. Partitioned polygenic score for type 2 diabetes susceptibility due to poor insulin secretion – INSPIRED & UK Biobank

ESM Table 5. Allele frequencies from variants in the insulin secretion/beta-cell function pPS in INSPIRED (ESDC and DMDSC) as well as UK Biobank cohorts. 5

**Gene**	**SNP**	**Chromosome**	**Risk Allele**	**RAF INSPIRED-DMDSC**	**RAF INSPIRED-ESDC**	**RAF UKB South Asians**	**RAF UKB white Europeans**	**Susceptibility Beta HOMA-B**
MTNR1B	rs10830963	11	G	0.34	0.28	0.32	0.25	-0.039
GCK	rs730497	7	A	0.11	0.19	0.13	0.17	-0.025
TCF7L2	rs7903146	10	T	0.30	0.32	0.29	0.3	-0.02
ADCY5	rs11708067	3	A	0.48	0.47	0.48	0.47	-0.016
SLC30A8	rs3802177	8	C	0.48	0.46	0.47	0.46	-0.016
HNF1A	rs1169288	12	C	0.34	0.31	0.31	0.27	-0.0056
TMEM258	rs102275	11	T	0.49	0.44	0.48	0.44	-0.0054
ABO	rs505922	9	C	0.3	0.29	0.35	0.27	-0.0017

RAF: Risk allele frequency, INSPIRED-DMDMSC: the Asian Indian DMDMSC cohort, INSPIRED-ESDC: white European ESDC cohort, UKB refers to UKBiobank, susceptibility to HOMA-B calculated from estimates provided by Dupuis et al. [5]

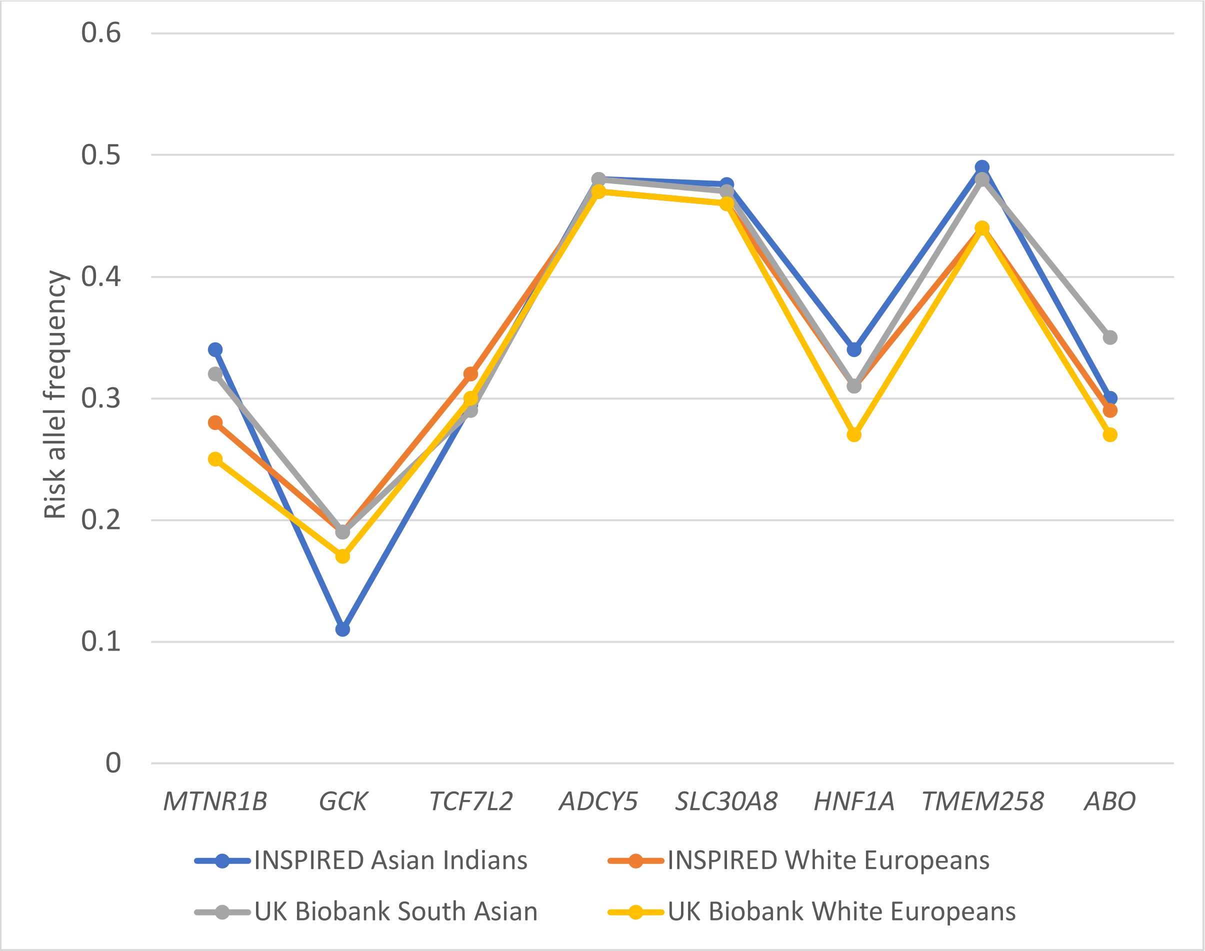

ESM Figure 5. Comparative risk allele frequencies (RAF) of insulin secretion/beta-cell function variants in the INSPIRED Asian Indian cohort (DMDSC – in blue) INSPIRED white European cohort (ESDC orange), UK Biobank South Asians (grey) and UK Biobank white Europeans (yellow). Variants are sorted in descending order from left to right based on effect size of susceptibility to poor beta cell function.

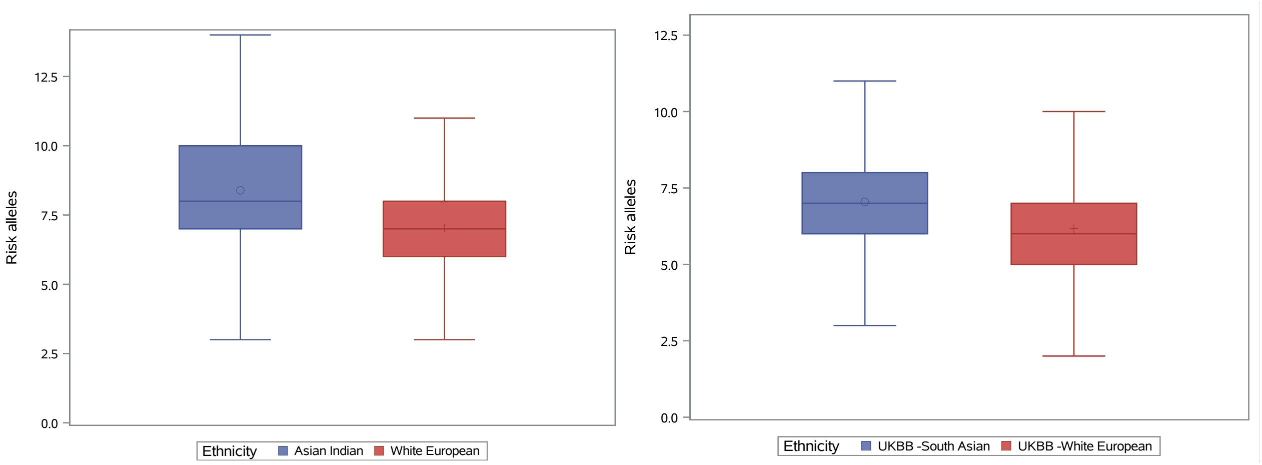

ESM Figure 6. Boxplots showing the difference in risk alleles for insulin secretion pPS in the INSPIRED cohort on the left. The difference in the empirical distribution of risk alleles between Asian Indians (n=5806, in blue) and white Europeans (n=6933, in red) was significant (Kolmogrov-Smirnov Pvalue <0.0001). Asian Indians had significantly more risk alleles (Wilcoxon-Mann-Whitney P value < 0.0001). Replication of difference in risk in UKBiobank on the right show the distribution of risk alleles of insulin secretion of pPS in White Europeans (n = 17,183 in red) and South Asians (n=1153 in blue) with type 2 diabetes. The empirical distribution of unweighted pPS also showed a significant difference using the Kolmogorov-Smirnov asymptotic two-sample test (P value<0.0001). South Asians had significantly more risk alleles (Wilcoxon-Mann-Whitney P value < 0.0001).

ESM Results 3. Weighted partitioned polygenic score – INSPIRED DMDSC

ESM Table 6. Test for normality of distributions for the beta-cell function weighted partitioned polygenic score in the INSPIRED study cohorts

Measures of tendencies	White European pPS	Asian Indian pPS
**N**	6933	5806
**Skewness**	-0.38	-0.13
**Kurtosis**	-0.19	-0.38
**Mean (SD)**	-0.062 (0.035)	-0.102 (0.036)
**Median (IQR)**	-0.059 (0.048)	-0.102 (0.048)
**Mode**	-0.016	-0.103

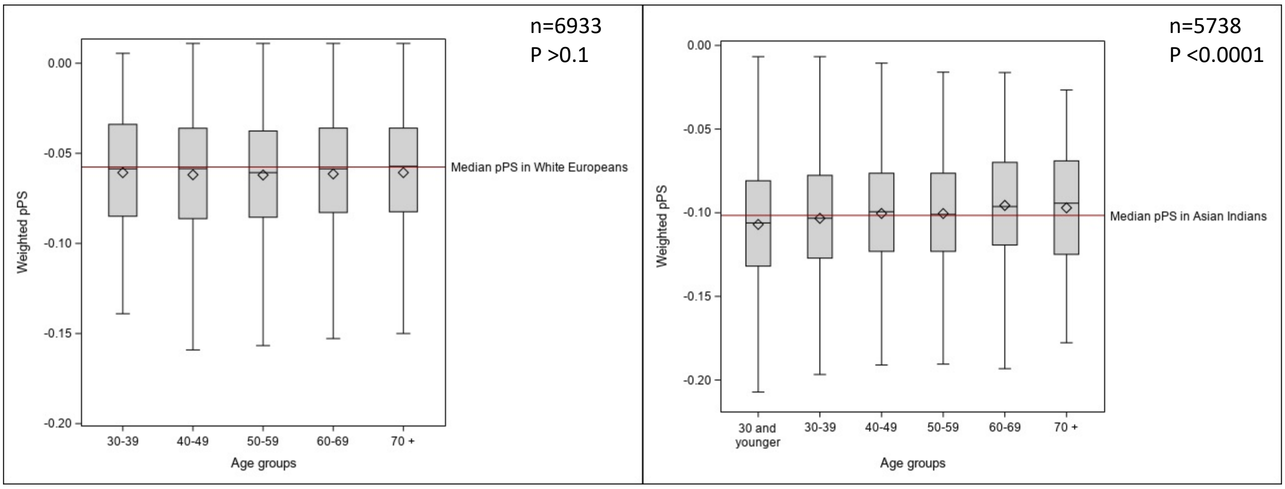

ESM Figure 7. Boxplots demonstrating the association between weighted beta cell function/insulin secretion pPs and age of diagnosis in white Europeans (left) and Asian Indians (right). Using Jonckheere-Terpstra test for trend, the Z statistics for white Europeans was non-significant, while for Asian Indians was 5.3, P value <0.0001. There were insufficient numbers of white Europeans diagnosed below the age of 30 years.

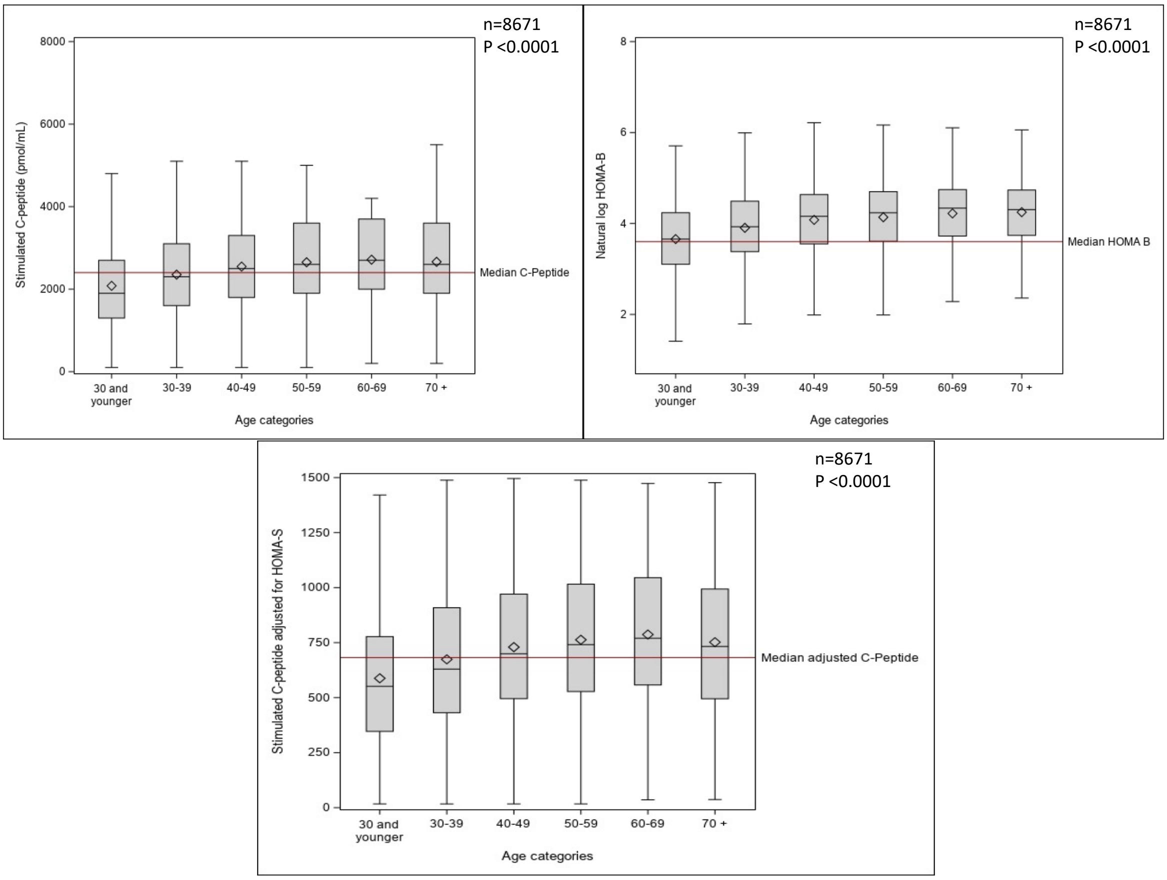

ESM Figure 8. Trend of stimulated C-peptide, HOMA-B and HOMA-B adjusted for insulin sensitivity with age groups at diagnosis in Asian Indians. Using Jonckheere-Terpstra test for trend, the Z statistics for stimulated C-peptides was 17.1 and P value < 0.0001, for HOMA B was 18.99 and P value < 0.0001 and for stimulated c-peptides adjusted for insulin sensitivity was 15.7 and P value <0.0001.

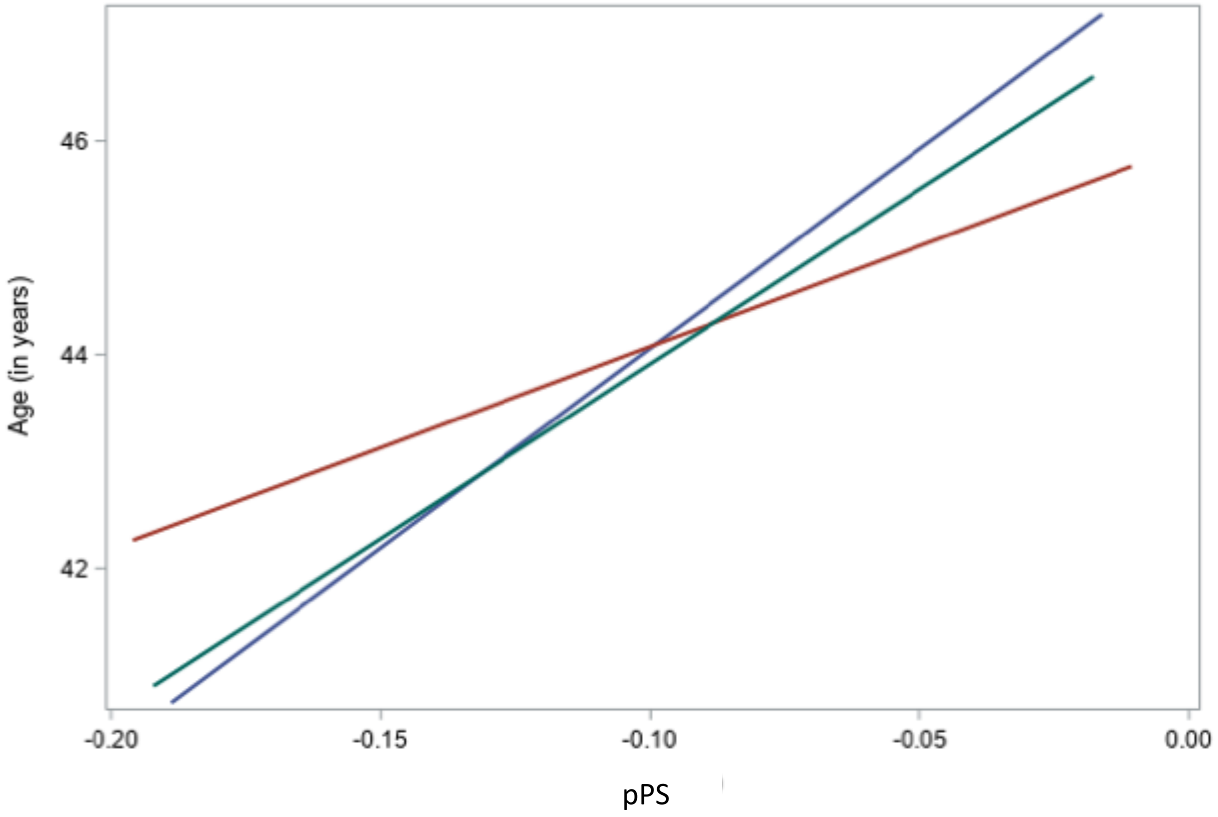

ESM Figure 9. Beta cell function partitioned polygenic risk score for beta cell function is a stronger predictor of age at diagnosis for those with normal BMI (in blue) compared to overweight (green) or obese BMI (red). Statistics provided in ESMTable 7.

ESM Table 7. Linear association of beta cell pPS and age at diagnosis of diabetes in Asian Indians stratified by BMI category.

**Sub-groups**	**n**	**Beta for HOMA-B weighted GRS (SE)**	**P value**	**R^2**
**Normal BMI**	409	37.31 (16.7)	0.02	1.2 %
**Overweight BMI**	257	32.68 (20.7)	0.11	0.97%
**Obese BMI**	847	18.9 (10.76)	0.08	0.36%

References

1. Hébert HL, Shepherd B, Milburn K, et al (2018) Cohort Profile: Genetics of Diabetes Audit and Research in Tayside Scotland (GoDARTS). Int J Epidemiol 47(2):380-381j. https://doi.org/10.1093/ije/dyx140
2. Anjana RM, Pradeepa R, Deepa M, et al (2011) The Indian Council of Medical Research-India Diabetes (ICMR-INDIAB) study: methodological details. J Diabetes Sci Technol 5(4):906–14. https://doi.org/10.1177/193229681100500413
3. Anjana RM, Deepa M, Pradeepa R, et al (2017) Prevalence of diabetes and prediabetes in 15 states of India: results from the ICMR–INDIAB population-based cross-sectional study. Lancet Diabetes Endocrinol 5(8):585–596. https://doi.org/10.1016/S2213-8587(17)30174-2
4. McCarthy S, Das S, Kretzschmar W, et al (2016) A reference panel of 64,976 haplotypes for genotype imputation. Nat Genet 48(10):1279–1283. https://doi.org/10.1038/ng.3643
5. Dupuis J, Langenberg C, Prokopenko I, et al (2010) New genetic loci implicated in fasting glucose homeostasis and their impact on type 2 diabetes risk. Nat Genet 42(2):105–116. https://doi.org/10.1038/ng.520
